# Cutaneous angiosarcoma of the scalp in a pediatric patient with xeroderma pigmentosum

**DOI:** 10.1016/j.jdcr.2023.08.039

**Published:** 2023-09-07

**Authors:** Valeria S. Oliver-García, Kevin J. Moore, Thomas Denize, Mai P. Hoang, Victor A. Neel, Shadmehr Demehri

**Affiliations:** a Department of Dermatology, Massachusetts General Hospital and Harvard Medical School, Boston, Massachusetts; b Department of Pathology, Massachusetts General Hospital and Harvard Medical School, Boston, Massachusetts

**Keywords:** cutaneous angiosarcoma, frozen section biopsy, Mohs micrographic surgery, xeroderma pigmentosum

## Introduction

Xeroderma pigmentosum (XP) is a rare, DNA repair defect syndrome that presents with significant sensitivity to UV exposure. Patients with XP have significantly increased risk of cutaneous tumors, specifically nonmelanoma and melanoma skin cancers.^1^^,^^2^ Cutaneous angiosarcoma is a rare, aggressive, vascular tumor that typically presents in sites of prior radiation or chronic UV exposure.^3^ Angiosarcoma in patients with XP presents a treatment conundrum as 1 of the main treatment approaches to angiosarcoma is radiation therapy, whereby the ionizing radiation poses a risk for further morbidity.^4^ Here, we present a case of a 17-year-old male with XP diagnosed with cutaneous angiosarcoma of the scalp.

## Case report

A 17-year-old male from the Dominican Republic with XP and past medical history of multiple nonmelanoma skin cancers was referred for evaluation of a new, enlarging lesion on the parietal scalp that had been present for 3 months. He denied fever, unintentional weight loss, or other systemic/constitutional symptoms. Owing to travel and economic barriers as well as a history of multiple nonmelanoma skin cancers, the patient was referred directly for frozen biopsy and subsequent planned Mohs micrographic surgery (MMS).

**Fig 1:**
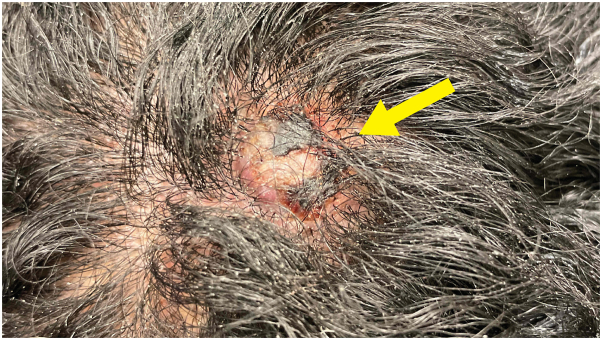
Hyperkeratotic nodule with hemorrhagic crust (*arrow*) on the left side of the posterior parietal scalp.

**Fig 2:**
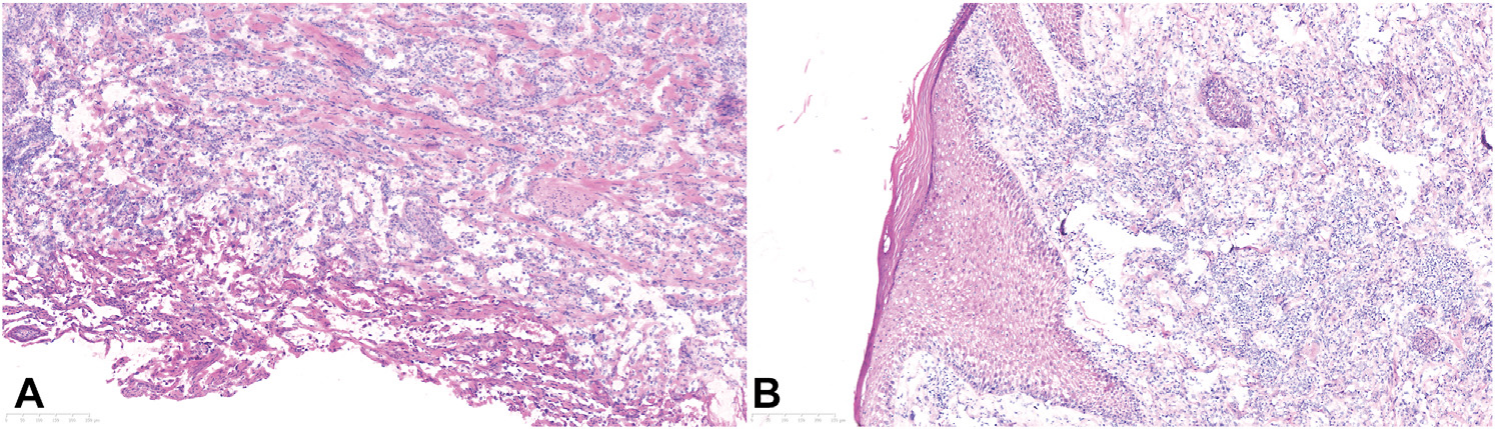
**A**, Frozen section of the scalp lesion biopsy. **B**, Bland monomorphic infiltrate with vascular element noted in the dermis.

**Fig 3:**
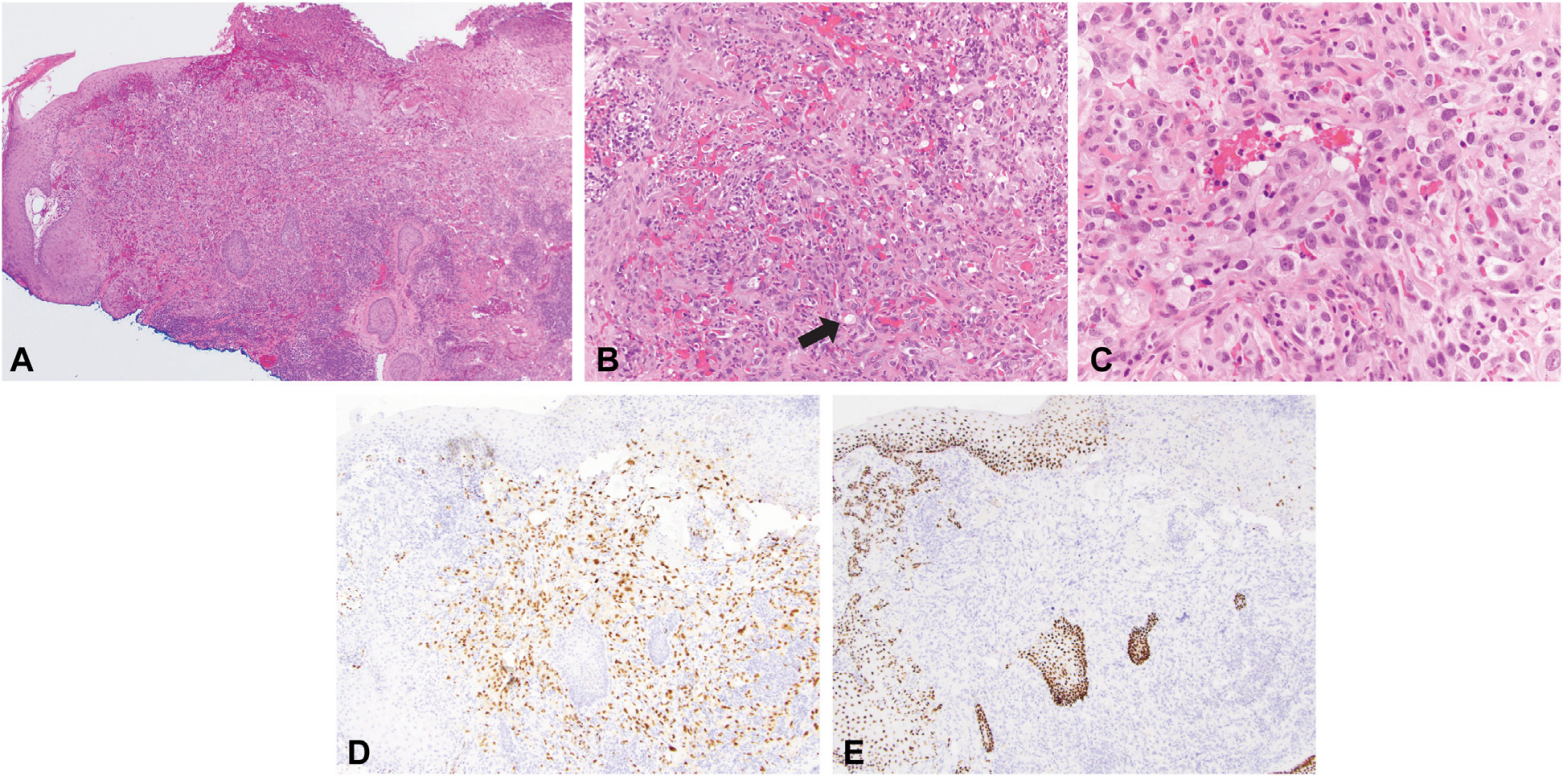
Paraffin-embedded permanent section of the scalp lesion. **A**, Low power image of hematoxylin-eosin–stained histologic section of the scalp lesion shows an ulcerated tumor (hematoxylin-eosin stain; original magnification: ×40). **B**, **C** High power images show markedly atypical epithelioid tumor cells forming vascular channels (*arrow*) (hematoxylin-eosin stain; original magnification: **B**, ×200; **C**, ×400). **D**, Erythroblast transformation-specific related gene immunostain—a vascular marker—strongly stains the tumor cells (original magnification: ×200). **E,** p40 stains the keratinocytes within the epidermis and hair follicles but not the tumor cells, excluding the possibility of a poorly differentiated squamous cell carcinoma (original magnification: ×200).

Clinical examination demonstrated a hyperkeratotic, firm 1.3 × 1.5-cm nodule with hemorrhagic crust on the left side of the posterior parietal scalp (Fig 1). A frozen biopsy was performed, which demonstrated a bland vascular neoplasm in the papillary dermis with a monomorphic infiltrate (Fig 2, *A* and *B*). Differential diagnosis included pyogenic granuloma vs malignant neoplasm. Given the inconclusive pathologic diagnosis on the frozen section, MMS was not performed, and a deeper shave biopsy sample was obtained and sent for paraffin-embedded permanent section processing with immunostaining. The deeper biopsy demonstrated atypical epithelioid cells forming vascular channels that stained positive for erythroblast transformation-specific related gene and negative for p40 and human herpes virus 8, confirming the diagnosis of angiosarcoma (Fig 3, *A* to *D*).

After the diagnosis of angiosarcoma, the patient was referred to Pediatric Hematology and Oncology and Head and Neck Surgical Oncology for further management. A staging positron emission tomography-computed tomography scan showed no evidence of locoregional spread or metastases. Owing to patient’s XP diagnosis, radiation therapy was not recommended. Head and neck surgeons performed a 3.5 × 3-cm excision using peripheral and deep en face margin analysis to assure clear surgical margins. The patient will continue active surveillance with regular imaging and clinical monitoring.

## Discussion

This report presents a rare case of cutaneous angiosarcoma in a pediatric patient with XP initially referred for frozen section biopsy and MMS for presumed nonmelanoma skin cancer. To the best of our knowledge, this is the twelfth case report of a pediatric patient with XP diagnosed with cutaneous angiosarcoma. Although not as common as nonmelanoma and melanoma skin cancers, angiosarcoma should be considered in patients with XP. Angiosarcoma tumors arise in sites of chronic UV exposure or prior irradiation, and patients with XP have an intrinsic defect in the DNA damage repair process. Clinical presentation of angiosarcoma varies, and it can present as a benign lesion or other malignant lesion, as seen in this case. Given the link between UV and radiation exposure and angiosarcoma, it is important to consider angiosarcoma in the differential for cutaneous tumors in patients with XP.

This case also highlights a potential pitfall in frozen section biopsy process before MMS. Prior retrospective studies have demonstrated high rates of accuracy in frozen section biopsy diagnoses for nonmelanoma skin cancers.^5^ In addition, Machan et al^6^ reported that the majority of patients prefer to have the biopsy and MMS in the same day. In this case, given the clinical exam and this patient’s history of multiple squamous cell carcinomas in the past, the squamous cell carcinomas diagnosis was presumed before frozen section. The patient’s economic and travel situation also warranted frozen section biopsy and same day MMS. Although nonmelanoma skin cancers have been proven to be ideal for frozen section processing, angiosarcoma is not ideal for frozen section processing because the classic dermal vessels and channels with malignant cells are collapsed and obscured by the freezing process.^7^ Although frozen section biopsy provides advantages in efficiency and patient satisfaction, this case highlights one of the limitations of the frozen section biopsy process for some pathologic diagnoses and the importance of immunostaining for diagnosis confirmation.

Surgical resection is the primary treatment approach for cutaneous angiosarcoma. However, angiosarcoma treatment should be individualized to the patient in terms of sociodemographic, clinical, and tumor characteristics. This case presents a challenging treatment quandary with an international pediatric patient with XP, raising concerns for treatment complications, specifically with radiation therapy.^4^ With no evidence of locoregional progression or metastases, the patient was managed with surgical resection and active surveillance. Case reports have demonstrated that patients with XP with angiosarcoma are treated successfully with surgery monotherapy.^8^ Of note, there have been case reports on patients with XP with aggressive nonmelanoma skin cancers treated with radiation therapy without significant complications.^9^ For patients with XP with advanced angiosarcoma, newer treatment modalities such as systemic immunotherapy have been shown to be successful.^10^ Patients with angiosarcoma, specifically medically complex patients with conditions such as XP, benefit from a multidisciplinary care team including dermatology, hematology/oncology, pathology, radiation oncology, and surgical oncology to ensure optimum and comprehensive care.

## Conflicts of interest

None.
